# Discovery of restless legs syndrome plasmatic biomarkers by proteomic analysis

**DOI:** 10.1002/brb3.1062

**Published:** 2018-09-22

**Authors:** Elisa Bellei, Emanuela Monari, Serkan Ozben, Mesrure Koseoglu Bitnel, Selma Topaloglu Tuac, Aldo Tomasi, Stefania Bergamini

**Affiliations:** ^1^ Department of Diagnostic and Clinical Medicine and Public Health Proteomic Lab University of Modena and Reggio Emilia Modena Italy; ^2^ Department of Neurology Antalya Training and Research Hospital Antalya Turkey; ^3^ Department of Neurology Bakirkoy Psychiatry and Neurology Research and Training Hospital Istanbul Turkey

**Keywords:** biomarkers, diagnosis, mass spectrometry, proteomics, restless legs

## Abstract

**Objectives:**

Restless legs syndrome (RLS) can lead to severe clinical consequences, thus negatively impacts on patients’ overall health and quality of life. Nevertheless, the pathophysiology of RLS is still unclear, resulting in underestimate, incorrect, or ignored diagnosis and in limited management and treatment. The aim of this study was to compare the plasma proteome of RLS patients and healthy controls, in the search of diagnostic biomarkers related to the disease severity.

**Materials and Methods:**

Two‐dimensional gel electrophoresis coupled with liquid chromatography‐mass spectrometry was employed to analyze plasma samples of 34 patients with primary RLS, divided into two subgroups according to the disease severity: MMS group (mild‐moderate symptoms) and HS group (severe and very severe symptoms), and 17 age‐ and sex‐matched control subjects. Sleep quality, daytime sleepiness, and the level of depression were also evaluated.

**Results:**

We identified eight upregulated spots, corresponding to five unique proteins, in both RLS group vs. controls (alpha‐1B‐glycoprotein, alpha‐1‐acid glycoprotein 1, haptoglobin, complement C4‐A, and immunoglobulin kappa constant); five increased spots, consistent with three unique proteins, only in HS‐RLS (kininogen‐1, immunoglobulin heavy constant alpha 1, and immunoglobulin lambda constant 2); one downregulated spot in both patient's groups (complement C3) and another one only in HS‐RLS (alpha‐1‐antitrypsin).

**Conclusions:**

The significantly different plasma proteins detected in RLS were mainly associated with inflammation, immune response, and cardiovascular disorders. Particularly, the gradual increasing in immunoglobulins could be indicative of the disease severity and evolution. Accordingly, these proteins may represent a valid set of useful biomarkers for RLS diagnosis, progression and treatment.

## 1 INTRODUCTION

Restless legs syndrome (RLS), also called Willis–Ekbom disease, is a neurological sensorimotor condition characterized by unpleasant sensations in the legs, which leads to the pressing need to move the limbs, thus relieving the associated discomforts, such as paresthesias and dysesthesias (Allen et al., 2003). These symptoms occurring at rest and their intensity increase in the evening or night, often inducing spontaneous episodic jolting of the legs that, in turn, cause serious sleep disruption and impairs quality of life (Trotti, 2017).

It is estimated that RLS prevalence is higher in Europe and North America, ranging from 5% to 10%, and lower in Asia, where it affects approximately 1%–7% of the adult population. Moreover, the prevalence increases with age and it is suggested that the syndrome is also associated with female sex, low socioeconomic status, smoking, frequent alcohol consumption, low education, and unhealthy lifestyle (Didriksen et al., 2017).

Additionally, recent studies have shown some risk factors connected to RLS, such as coronary heart disease (Li et al., 2012) and other complications that can cause or worsen RLS, for example, diabetic neuropathy (Gemignani, Brindani, & Marbini, 2008), chronic kidney disease(Novak, Winkelman, & Unruh, 2015), and iron deficiency (Connor, 2008), as well as various consequences of the syndrome, such as insomnia, anxiety, and depression (Earley & Silber, 2010).

To date, the pathophysiology of primary RLS is still largely unknown, although dysfunctions in the dopaminergic system are suspected to have a role in its development. In support of this assumption is that the current agents of choice for moderate‐to‐severe RLS are non‐ergot‐derived dopamine receptor agonists, even if augmentation is a known complication of dopaminergic medications (Nagandla & De, 2013).

Although RLS has gained notable consideration in the last 20 years, the diagnosis is still predominantly based on clinical features and the use of self‐administered questionnaires, focused on the four essential diagnostic criteria, that however are insufficient for a reasonably valid and complete RLS assessment (Allen et al., 2003). Nowadays, there are no standardized diagnostic tests for RLS; poor recognition or strange description of symptoms often causes delay in the diagnosis or, even worse, in misdiagnosis of RLS, resulting in undertreatment which, consequently, can leads to severe risk of metabolic deregulation, autonomic dysfunction, and cardiovascular morbidity (Nagandla & De, 2013). Therefore, a correct diagnosis and an appropriate management of RLS are very important. Particularly, to further prevent morbidity, RLS should be diagnosed in early stage. In this way, a valid help for the identification of novel diagnostically relevant biomarkers can derive from proteomic discovery strategies (Crutchfild, Thomas, Sokoll, & Chan, 2016). To our knowledge, only one proteomic study was previously conducted to search RLS biomarkers in the cerebrospinal fluid (CSF) of RLS patients, identifying six candidate protein markers for the early‐onset of RLS (Patton et al., 2013). An optimal biomarker should be highly sensitive and specific, as well as easily attainable, with a not invasive, or limited, procedure (Kohn, Azad, Annunziata, Dhamoon, & Whiteley, 2007). CSF sample collection requires a more complex and potentially dangerous procedure than blood, that, moreover, represents a reasonable source of biomarkers because it is directly exposed to all organs of the body and thus can be an archive of all ongoing processes.

The primary objective of our study was to analyze the plasma of RLS patients in comparison with healthy control subjects, in the search of potential diagnostic biomarkers of RLS, also considering the possible association with the disease severity. We applied proteomics methods based on two‐dimensional gel electrophoresis (2‐DE) coupled to liquid chromatography‐mass spectrometry analysis.

## 2 MATERIALS AND METHODS

### 2.1 Patients and controls selection

Primary RLS patients (*n* = 34) and healthy subjects as control group (*n* = 17) from the medical staff were recruited at the Neurology Department of Bakirkoy Psychiatry and Neurology Research and Training Hospital Sleep Disorders Center (Istanbul, Turkey).

For all study participants, exclusion criteria were as follows: systemic or neurologic diseases, other diagnosed sleep disorders, anemia, iron deficiency, uremia, thyroid disorders, use of antidepressants and antipsychotics, excessive alcohol intake.

All patients were diagnosed as primary RLS according to the International Classification of Sleep Disorders diagnostic criteria (American Academy of Sleep Medicine, 2014), while the degree of RLS severity was evaluated, according to the 10‐item International Restless Legs Syndrome Study Group (IRLSSG) rating scale, as follows: mild (IRLS scores, 1–10), moderate (IRLS scores, 11–20), severe (IRLS scores 21–30), and very severe (IRLS scores 31–40) (Allen et al., 2014).

Based on the results, RLS patients were divided into two subgroups: low‐medium severity (MMS) group, (*n* = 17) including all patients with mild and moderate IRLS scores, and high severity (HS) group (*n* = 17), comprising patients with severe and very severe IRLS scores (Table 1). Patients and control groups were strictly matched for age and gender, as shown in Table 1.

**Table 1:** Demographic and clinical data of RLS patients and controls

	Controls (*n* = 17)	MMS‐RLS (*n* = 17)	HS‐RLS (*n* = 17)	*p*‐Value
Age	44.5 ± 7.0	45.1 ± 11.0	44.6 ± 6.9	0.941^a^
				0.896^b^
Gender				
Male	5 (29.4%)	7 (41.2%)	5 (29.4%)	1.000^a^
Female	12 (70.6%)	10 (58.8%)	12 (70.6%)	0.488^b^
RLS onset				
<3months	—	2 (11.8%)	1 (5.9%)	0.463^b^
3months–1year	—	2 (11.8%)	1 (5.9%)	
1–5years	—	5 (29.4%)	5 (29.4%)	
>5years	—	8 (47.0%)	10 (58.8%)	
Iron parameters				
Hemoglobin (g/dl)	—	13.9 ± 1.3	13.7 ± 1.2	0.578^b^
Hematocrit (%)	—	41.4 ± 6.0	41.6 ± 3.6	0.923^b^
Mean cell volume (fl)	—	86.6 ± 4.1	85.0 ± 3.5	0.241^b^
Serum iron (μg/dl)	—	113.3 ± 69	78.7 ± 30.8	0.069^b^
Ferritin (μg/L)	—	83.0 ± 51.7	68.9 ± 38.7	0.395^b^

*Notes*

Age data and iron parameters are expressed as mean ± standard deviation.

*p*‐Value based on the Student's *t* test: ^a^HS‐RLS vs. controls; ^b^HS‐RLS vs. MMS‐RLS group.

The study received the approval of the local Ethical Committee and was carried out in conformity with the Helsinki Declaration. Informed consent to the study was provided by each subject.

### 2.2 Patients rating scales

For all RLS patients were also evaluated: (a) the level of depression by the Beck Depression Inventory Score (Beck, Steer, & Carbin, 1988); (b) the quality and patterns of sleep by the Pittsburgh Sleep Quality Index (PSQI) (Buysse, Reynolds, Monk, Berman, & Kupfer, 1989); (c) the daytime sleepiness and tendency to sleep during the day by the Epworth Sleepiness Scale (ESS; Izci et al., 2008).

### 2.3 Plasma samples collection and treatment

Fasting morning venous blood was collected from both RLS patients and controls into EDTA tubes. Plasma was separated by centrifugation, aliquoted, and stored frozen at −80°C until use. Before proteomic analysis, plasma samples from each of the three groups (controls, MMS‐RLS and HS‐RLS) were combined to form three different pools per group.

Moreover, in biomarkers discovery studies, depletion strategies are necessary to decrease the high dynamic concentration range of plasma proteome, allowing the detection of the low‐abundance component fraction, even if this could lead to the concomitant elimination of nontargeted proteins (Bellei et al., 2011).

In this work, the complexity of plasma sample was reduced by the removal of albumin and IgG, the two most abundant plasma proteins, using the ProteoPrep^**®**^ Immunoaffinity Albumin and IgG Depletion Kit (Sigma), that specifically removes these proteins by an immunodepletion method (Bellei et al., 2011). Finally, total protein content was measured spectrophotometrically at λ 595 nm.

### 2.4 Two‐dimensional gel electrophoresis (2‐DE)

Protein separation was performed on depleted plasma samples by 2‐DE, as previously described (Bellei et al., 2017). Briefly, 80 μg of protein was diluted with rehydration buffer and then loaded onto 7‐cm‐long immobilized pH gradient strips (Ready IPG Strip^™^, Bio‐Rad), pH range 3–10. Afterward, the strips were first subjected to isoelectric focusing and then to the second‐dimension separation on 10% polyacrylamide gels, stained with Coomassie Blue. The gel images were acquired by a calibrated densitometer (GS‐800, Bio‐Rad) and analyzed by the PDQuest 2‐D image analysis software (Bio‐Rad), as previously reported (Bellei et al., 2017).

### 2.5 Mass spectrometry analysis and protein identification

The differentially expressed protein spots were “in‐gel” digested with trypsin, to obtain peptides mixture to be analyzed through MS, as previously described (Bellei et al., 2011). The concentrated peptides were analyzed by a LC‐Chip‐MS System, composed of the Electrospray Ionization Quadrupole‐Time of Flight Mass Spectrometer (ESI‐Q‐ToF‐MS) (Accurate‐Mass G6520, Agilent Technologies) connected to a Nano HPLC‐Chip microfluidic device (1200 Nano HPLC‐Chip, Agilent Technologies). The MASCOT search engine (http://mascot.cigs.unimo.it/mascot), together with the UniProt knowledgebase database, was used for peptide sequence searching. The highest score hits among MASCOT search results were selected, and proteins were considered identified with at least two unique peptides. Moreover, protein identification was repeated at least once, using spots extracted from different gel.

### 2.6 Statistical analysis

The Student's *t* test was used to compare demographic and clinical data of RLS patients and control subjects, considering a *p*‐value <0.05 as statistically significant. All reported data are expressed as mean ± standard deviation (*SD*).

## 3 RESULTS

### 3.1 Patients and controls data

Demographic information about RLS patients and controls are provided in Table 1. As evidenced, no significant differences concerning age and gender were detected comparing RLS and control groups.

According to the severity of symptoms assessed by the IRLSSG rating scale for RLS, patients were divided into two subgroups: mild‐moderate severity (MMS) group (17 patients, 50%) and high severity (HS) group (17 patients, 50%). In Table 1 is also reported the RLS duration, namely the onset age of RLS symptoms, that was >5 years for the majority of patients, in both RLS groups (47% for MMS group and 58.8% for HS group, respectively).

**Figure 1:**
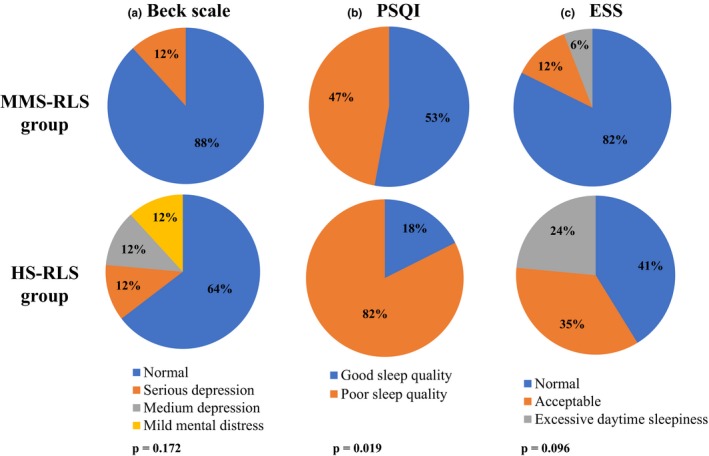
Pie charts reporting the scores obtained from the rating scales. The scores achieved for MMS‐RLS and HS‐RLS groups are reported in percentage: (a) Beck Depression Inventory Score, (b) Pittsburgh Sleep Quality Index (PSQI), (c) Epworth Sleepiness Scale (ESS). *p*‐Value obtained by the Student's *t* test

The results obtained from the patients rating scales are illustrated in Figure 1. According to the Beck Depression Inventory Scale (Figure 1a), 15 MMS‐RLS patients (88%) were without depression symptoms and only two (12%) presented serious depression, while in HS group the 36% of patients showed mild‐serious depression. PSQI scores (Figure 1b) were significantly higher in HS group compared to MMS group (*p* = 0.019), indicating that HS‐RLS patients had worse overall sleep quality. Finally, according to the ESS (Figure 1c), only one MMS‐RLS patient (6%) received >10 points, reporting excessive daytime sleepiness, compared to the 24% (four patients) of HS group.

### 3.2 Bidimensional gel electrophoresis and mass spectrometry analysis

**Figure 2:**
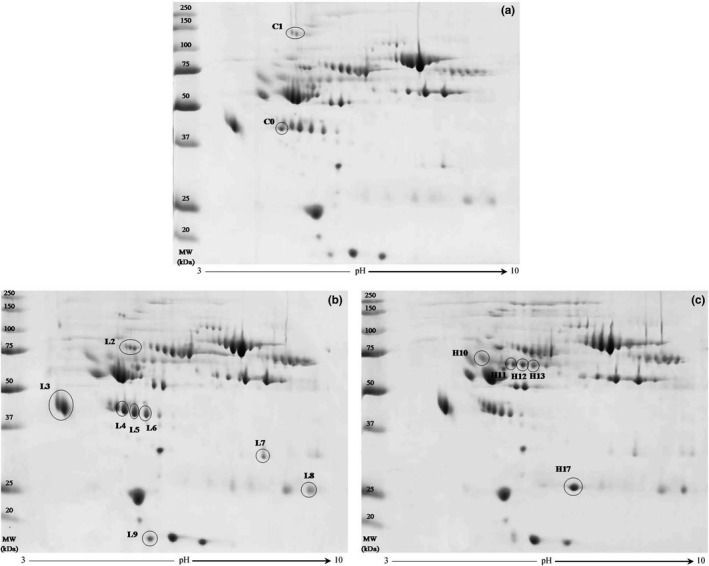
Representative 2D gel maps. (a) Control group, (b) MMS‐RLS patients group, (c) HS‐RLS patients group. In gel images are marked the protein spots significantly different among the three groups, identified by ESI‐Q‐ToF‐MS. Panel (a) shows the decreased protein spots, panel (b) illustrates the spots increased in both RLS groups vs. controls, and panel (c) the spots increased in HS‐RLS group vs. MMS‐RLS and control groups. Alphanumeric codes in the figures correspond to those listed in Table 2. Each gel map reports the molecular weight (MW) standard protein ladder, expressed in kilodalton (kDa) (All Blue Precision Plus Protein^™^ Standards, Bio‐Rad). First dimension separation: IPG strip, pH 3–10. Second‐dimension separation: 10% polyacrylamide gels

The 2D‐gel maps were analyzed by the PDQuest image analysis software, after a normalization step to correct the variability due to the staining process. A total of 15 protein spots were found differentially expressed between RLS and controls (Figure 2). All the selected spots were identified by ESI‐Q‐ToF‐MS analysis; the characterized proteins are listed in Table 2, wherein the alphanumeric annotation of each spot corresponds to that reported in Figure 2.

**Table 2:** Differentially expressed plasma proteins in RLS identified by ESI‐Q‐ToF‐MS analysis

Spot ID	Entry name^a^	Acc. number^b^	Gene name	MW (Da)	Score^c^	Peptide match/sig. match^d^	Seq/sig.seq^e^	Seq. cov. (%)^f^	emPAI^g^	Fold change^h^
Protein downregulated in both RLS groups										
C0	CO3	P01024	C3	188,569	3,219	192/123	34/27	18	0.73	−3.20#
Protein downregulated in HS‐RLS										
C1	A1AT	P01009	SERPINA1	46,878	1,280	115/58	27/18	56	3.26	−4.07#
Proteins upregulated in both RLS groups										
L2	A1BG	P04217	A1BG	54,790	793	69/45	18/13	44	1.17	+1.57*
L3	A1AG1	P02763	ORM1	23,725	1,210	74/49	13/10	49	3.56	+1.51*
L4	HPT	P00738	HP	45,861	693	93/47	17/14	39	1.85	+2.27*
L5	HPT	P00738	HP	45,861	1,037	164/70	20/15	42	3.67	+2.63*
L6	HPT	P00738	HP	45,861	1,383	149/73	20/16	36	2.43	+2.81*
L7	CO4A	P0C0L4	C4A	194,261	1,390	150/82	16/11	7	0.23	+1.50*
L8	IGKC	P01834	IGKC	11,773	1,573	66/55	7/7	88	8.60	+2.34*
L9	HPT (frag.)	P00738	HP	45,861	443	26/17	11/6	20	0.54	+1.66*
Proteins upregulated in HS‐RLS										
H10	KNG1	P01042	KNG1	72,996	834	81/52	26/20	34	1.36	+5.36#
H11	IGHA1	P01876	IGHA1	38,486	1,253	81/48	15/12	44	1.99	+2.54#
H12	IGHA1	P01876	IGHA1	38,486	1,144	83/44	15/12	44	1.40	+2.90#
H13	IGHA1	P01876	IGHA1	38,486	1,066	74/42	14/10	44	1.40	+3.72#
H17	IGLC2	P0DOY2	IGLC2	11,458	1,527	70/51	6/5	69	4.08	+10.64#

^a^Protein entry name (with extension_HUMAN, UniProtKB database): CO3, complement C3; A1AT, alpha‐1‐antitrypsin; A1BG, alpha‐1B‐glycoprotein; A1AG1, alpha‐1‐acid glycoprotein 1; HPT, haptoglobin; CO4A, complement C4‐A; IGKC, immunoglobulin kappa constant; KNG1, kininogen‐1; IGHA1, immunoglobulin heavy constant alpha 1; IGLC2, immunoglobulin lambda constant 2. ^b^Primary accession number (UniProtKB database). ^c^The highest ion scores from MASCOT search engine. ^d^Total number of peptides matching the identified proteins/Significant matches. ^e^Total number of sequences/number of significant sequences. ^f^Sequence coverage: percentage of sequenced amino acids for the detected protein. ^g^Exponentially modified protein abundance index. ^h^Fold change of protein signal: level of protein spot expression change calculated as ratio between the spot intensity values in: ^#^HS‐RLS or *MMS‐RLS vs. controls. (−) decrease in RLS, (+) increase in RLS.

Notably, the great majority of spots (*n* = 13) was recognized as increased in RLS groups compared to controls, while only two spots resulted decreased. Specifically, of the 13 upregulated spots, eight resulted increased in both RLS patient's groups, and corresponded to five unique proteins: alpha‐1B‐glycoprotein (A1BG), alpha‐1‐acid glycoprotein 1 (A1AG1), haptoglobin (HPT), complement C4‐A (CO4A), and immunoglobulin kappa constant (IGKC). In Table 2 is reported only the fold change value calculated in MMS‐RLS/controls, because those obtained from HS‐RLS/controls were very similar, without significant differences.

Five spots, corresponding to three unique proteins, were found upregulated only in HS‐RLS group: kininogen‐1 (KNG1), immunoglobulin heavy constant alpha 1 (IGHA1), and immunoglobulin lambda constant 2 (IGLC2); this latter with the higher expression change vs. MMS‐RLS (+10.64), as shown in Table 2. Finally, one spot resulted downregulated in both RLS groups, identified as complement C3 (CO3), and another one only in HS‐RLS, recognized as alpha‐1‐antitrypsin (A1AT).

## 4 DISCUSSION

Nowadays, the causes of RLS are not yet completely known and this makes both the diagnosis and the therapeutic approach quite difficult. There is a great need for intensive basic and clinical research to discover new and reliable biomarkers, useful firstly for a proper diagnosis and afterward to develop more effective and specific medications to treat RLS symptoms.

In the present study, by a proteomic approach, we found an expression level of 15 protein spots, corresponding to 10 unique proteins, significantly altered in plasma of RLS patients.

Specifically, one protein, A1AT, resulted decreased in HS‐RLS group (Figure 2 and Table 2). Literature data show a correlation between A1AT deficiency and CVD, in addition to musculoskeletal comorbidities (Duckers et al., 2010; Fähndrich et al., 2017). Circulating A1AT has a protective role in limitating vascular damage, other than to be involved in the regulation of vascular smooth muscle cells and control of inflammatory pathways. Low levels of A1AT, leading to unopposed neutrophil elastase activity in the vascular system, may cause the local degradation of elastin and the consequent increased collagen deposition. This condition, especially in larger central arteries, may induce arterial stiffness, with risk to develop arteriosclerosis (Duckers et al., 2010). Our findings could suggest a greater risk of CVD especially in HS‐RLS patients, as the expression level of A1AT was four times lower compared to controls (Table 2).

Another protein, CO3, was found downregulated in both RLS groups vs. controls. Complement acts as a rapid and efficient immune surveillance system, with different effects on both healthy and altered host cells, other than to be involved in homeostasis control and in inflammatory processes (Ricklin, Hajishengallis, Yang, & Lambris, 2010). In consequence, defective control of the complement cascade, or of its regulation, is associated with several disorders. A decrease of CO3 in RLS patients could substantially contribute to worsening the inflammatory state associated with RLS. In support of this assumption is the further finding that two acute phase proteins, HPT and A1AG1, have been found overexpressed in both RLS patient's groups. As evident in Figure 2, spots L4‐L6 (different isoforms of the same protein), and spot L9 (derived from protein fragmentation), were identified as HPT by MS. HPT plasma concentration increases several folds in case of inflammatory stimulus; therefore, it is strongly associated with diseases that have inflammatory sources (Quaye, 2008). Moreover, elevate HPT levels have been observed in CSF of patients with peripheral neuropathy that causes acute neuromuscular failure, and in case of neuromyelitis optica, an idiopathic flogistic demyelinating disease of the CNS predominantly affecting optic nerves and the spinal cord (Bai et al., 2009).

A1AG1, or orosomucoid, is a glycoprotein which serum concentration rises several folds in response to local inflammatory stimuli, systemic tissue injury, or infection (Fournier, Medjoubi‐N, & Porquet, 2000). Its biological function is still not completely established, although, as a member of the lipocalin family, it has binding and transport activity, as well as immunomodulatory and anti‐inflammatory roles (Hochepied, Berger, Baumann, & Libert, 2003). The overexpression of A1AG1 and HPT observed in plasma of RLS patients could suggest that these subjects are more sensitive to inflammation, and our findings may be a response to the inflammatory complaint occurring in this syndrome, suggesting the possibility that RLS may be mediated, or negatively affected, by a condition of excessive inflammation. In a proteomic study performed on CSF, Patton et al. (2013) have found downregulated levels of A1AG, which reflect the altered iron homeostasis and the reduction of its content in the CNS of RLS patients.

In our work, other three proteins, A1BG, CO4A, and IGKC, resulted upregulated in both RLS group vs. controls, independently from the disease severity score (Table 2). A1BG is a secreted plasma protein and a member of the immunoglobulin superfamily with a reasonable role in the immune system and in cell adhesion (Halaby & Mornon, 1998). In literature are reported conflicting data concerning the expression of A1BG. For example, in neuromyelitis optica has been found a reduced expression of A1BG in CSF of patients compared to controls (Bai et al., 2009). Conversely, in case of autoimmune disorders, which frequently are associated with RLS, Biswas et al. (2013)have identified, by an immunoproteomic approach, a higher expression of A1BG in synovial fluid of patients with rheumatoid arthritis (RA), proposing this glycoprotein as a biomarker of diagnostic importance for RA.

CO4A has a central role in the activation of the classical pathway of the complement system. Moreover, it induces the contraction of smooth muscle and increases vascular permeability, other than to be a mediator of local inflammatory processes. This further strengthens our assumption of a great and significant presence of inflammation in RLS. A recent proteomic study ascribes to the increase of CO4A in CSF a negative prognostic role for multiple sclerosis, a pathology often associated with RLS (Füvesi et al., 2012).

Regarding IGKC, it was reported increased in serum of patients with autoimmune diseases, such as rheumatoid arthritis (Gottenberg et al., 2007) and multiple sclerosis (Kaplan, Golderman, Yahalom, Yaskariev, & Sela, 2013), and in CSF of subjects with specific CNS diseases, like neuromyelitis optica (Bai et al., 2009).

In addition to IGKC, we found other increased immunoglobulins, IGHA1 e IGLC2, but only in HS‐RLS group compared to MMS‐RLS and controls (Figure 2, Table 2). High levels of immunoglobulins impact on processes concerning the immune system, so the enhancing expression of immunoglobulins in RLS at high severity seems to indicate a progressively greater involvement of immunological mechanisms with the worsening of the disease. Noteworthy, as evident in Table 2, IGLC2 showed the higher level of protein spot expression change vs. MMS‐RLS group (+10.64). A massive upregulation of Ig‐related genes was also observed by immunohistochemistry in cortical sections of patients with multiple sclerosis (Torkildsen et al., 2010).

Finally, another protein increased only in HS group was KNG1. This is a major component of plasma kallikrein‐kinin system and a bradykinin precursor, a nanopeptide with strong vasoactive and proinflammatory properties. Studies on KNG1‐deficients mice demonstrated an important role of this protein in the pathogenesis of autoantibody‐induced arthritis, proposing the block of KNG1 cleavage as a novel therapeutic strategy for rheumatoid arthritis treatment (Xie, Dai, & Wu, 2016). A study conducted in mice recognized KNG1 as a key mediator of ischemic neurodegeneration and neuronal damage by increasing microvascular thrombosis, blood–brain barrier leakage and inflammation; KNG1 inhibition allowed to protect from thromboembolic disorders and stroke (Langhauser et al., 2012). In humans, extreme levels of high‐molecular‐weight kininogen can potentiate the risk of myocardial infarction and ischemic stroke (Siegerink, Rosendaal, & Algra, 2012). Our finding of increased KNG1 amount in HS‐RLS patients provides a further support, together with the downregulation of A1AT, for a greater risk of CVD, especially in RLS at high severity. This is in line with the literature, which proves the presence of a close relationship between vascular risk factors, CVD and RLS (Schlesinger, Erikh, Avizohar, Sprecher, & Yarnitsky, 2009; Winter et al., 2013). Moreover, it is well known that inflammation plays a larger role in the predisposition to CVD and that stroke may be mediated by inflammatory mechanisms. Considered all together, our findings strongly support and consolidate the theoretical roles of inflammatory mechanisms and immunological alterations proposed by Weinstock, Walters, & Paueksakon (2012) for the pathogenesis of RLS.

In the last few years, substantial progress has been made also in understanding the genetic basis of RLS, with focus on the identification of genes that may give rise to the disease (Winkelmann et al., 2017). Genomewide association studies (GWAS), meta‐analysis, twin, and family large‐scale studies have successfully contributed to identify significant association between RLS and common variants in MEIS1, BTBD9, and MAP2K5 genes (Thireau et al., 2017; Winkelmann et al., 2007). It should be noted that our patients were not sequenced for these allelic variations that however might be of relevant interest for the study.

Moreover, another important consideration is that 13 of 17 patients in the MMS group, and 15 of 17 patients in HS group, were under treatment with pramipexole (0.25–0.75 mg/day) before collecting blood samples, and only one patient in the HS group was taking also pregabalin (150 mg/day). We believe that medications could have an effect on proteomic analysis; hence, further studies may enlighten the impact of pharmacological treatment on the plasmatic proteome.

Finally, relatively to the rating scales used in the study, we found a significantly poorer sleep quality in HS‐RLS vs. MMS group, and a trend toward a higher daytime sleepiness, along with a greater propensity toward symptoms of medium‐severe depression (Figure 1).

## 5 CONCLUSIONS

In summary, the proteomic analysis of plasma from RLS patients revealed a defined protein cluster related to the involvement of biochemical networks principally linked to inflammatory and immune response; specifically, the increasing levels of immunoglobulins may account for the progression and severity of the disease. Moreover, the underexpression of A1AT and the upregulation of KNG1 are reflective of a greater risk for HS‐RLS patients to develop CVD; these proteins may represent potential early diagnostic biomarkers for cardiovascular and autoimmune disorders. Altogether, our data could suggest additional insights into the multifactorial pathophysiology of the RLS and contribute to enhance the inflammatory/immunological hypothesis proposed for the evolution of RLS process.

The proteomic analysis of plasma allowed a greater understanding of RLS, providing relevant information for its diagnosis and prognosis by the identification of a promising platform of candidate protein biomarkers, which could also be considered as helpful drug targets to develop innovative treatment strategies.

This is a preliminary mass spectrometry‐based proteomics study conducted for the first time on plasma samples from RLS patients, so the present results need to be confirmed and extended by increasing the number of cases, also applying complementary validation techniques (such as western blot and ELISA test).

## CONFLICT OF INTERESTS

All authors certify that they have no conflict of interests.
